# Ten-year all-cause death following percutaneous or surgical revascularization in patients with prior cerebrovascular disease: insights from the SYNTAX Extended Survival study

**DOI:** 10.1007/s00392-020-01802-x

**Published:** 2021-01-30

**Authors:** Rutao Wang, Kuniaki Takahashi, Scot Garg, Daniel J. F. M. Thuijs, Arie Pieter Kappetein, Michael J. Mack, Marie-Claude Morice, Friedrich-Wilhelm Mohr, Nick Curzen, Piroze Davierwala, Milan Milojevic, Robert Jan van Geuns, Stuart J. Head, Yoshinobu Onuma, David R. Holmes, Patrick W. Serruys

**Affiliations:** 1 Department of Cardiology, Xijing Hospital, Xi’an, China; 2 Department of Cardiology, National University of Ireland Galway, University Road, Galway, H91 TK33 Ireland; 3 Department of Cardiology, Radboud UMC, Nijmegen, The Netherlands; 4 Department of Cardiology, Amsterdam University Medical Center, University of Amsterdam, Amsterdam, The Netherlands; 5 East Lancashire Hospitals NHS Trust, Blackburn, Lancashire UK; 6 Department of Cardiothoracic Surgery, Erasmus University Medical Centre, Rotterdam, The Netherlands; 7 Department of Cardiothoracic Surgery, Baylor University Medical Center, Dallas, TX USA; 8 Département of Cardiologie, Hôpital Privé Jacques Cartier, Générale de Santé Massy, Massy, France; 9 University Department of Cardiac Surgery, Heart Centre Leipzig, Leipzig, Germany; 10 Cardiology Department, University Hospital Southampton, Southampton, UK; 11 Department of Cardiac Surgery and Cardiovascular Research, Dedinje Cardiovascular Institute, Belgrade, Serbia; 12 Department of Cardiovascular Diseases and Internal Medicine, Mayo Clinic, Rochester, MN USA; 13 NHLI, Imperial College London, London, UK

**Keywords:** Cerebrovascular disease, CABG, Left main coronary artery disease, PCI, Three-vessel disease

## Abstract

**Background:**

Coronary bypass artery grafting (CABG) has a higher procedural risk of stroke than percutaneous coronary intervention (PCI), but may offer better long-term survival. The optimal revascularization strategy for patients with prior cerebrovascular disease (CEVD) remains unclear.

**Methods and results:**

The SYNTAXES study assessed the vital status out to 10 year of patients with three-vessel disease and/or left main coronary artery disease enrolled in the SYNTAX trial. The relative efficacy of PCI vs. CABG in terms of 10 year all-cause death was assessed according to prior CEVD. The primary endpoint was 10 year all-cause death. The status of prior CEVD was available in 1791 (99.5%) patients, of whom 253 patients had prior CEVD. Patients with prior CEVD were older and had more comorbidities (medically treated diabetes, insulin-dependent diabetes, metabolic syndrome, peripheral vascular disease, chronic obstructive pulmonary disease, impaired renal function, and congestive heart failure), compared with those without prior CEVD. Prior CEVD was an independent predictor of 10 year all-cause death (adjusted HR: 1.35; 95% CI: 1.04–1.73; *p* = 0.021). Patients with prior CEVD had a significantly higher risk of 10 year all-cause death (41.1 vs. 24.1%; HR: 1.92; 95% CI: 1.54–2.40; *p* < 0.001). The risk of 10 year all-cause death was similar between patients receiving PCI or CABG irrespective of the presence of prior CEVD (p_-interaction_ = 0.624).

**Conclusion:**

Prior CEVD was associated with a significantly increased risk of 10 year all-cause death which was similar in patients treated with PCI or CABG. These results do not support preferential referral for PCI rather than CABG in patients with prior CEVD.

*Trial registration:* SYNTAX: ClinicalTrials.gov reference: NCT00114972. SYNTAX Extended Survival: ClinicalTrials.gov reference: NCT03417050.

**Graphic abstract:**

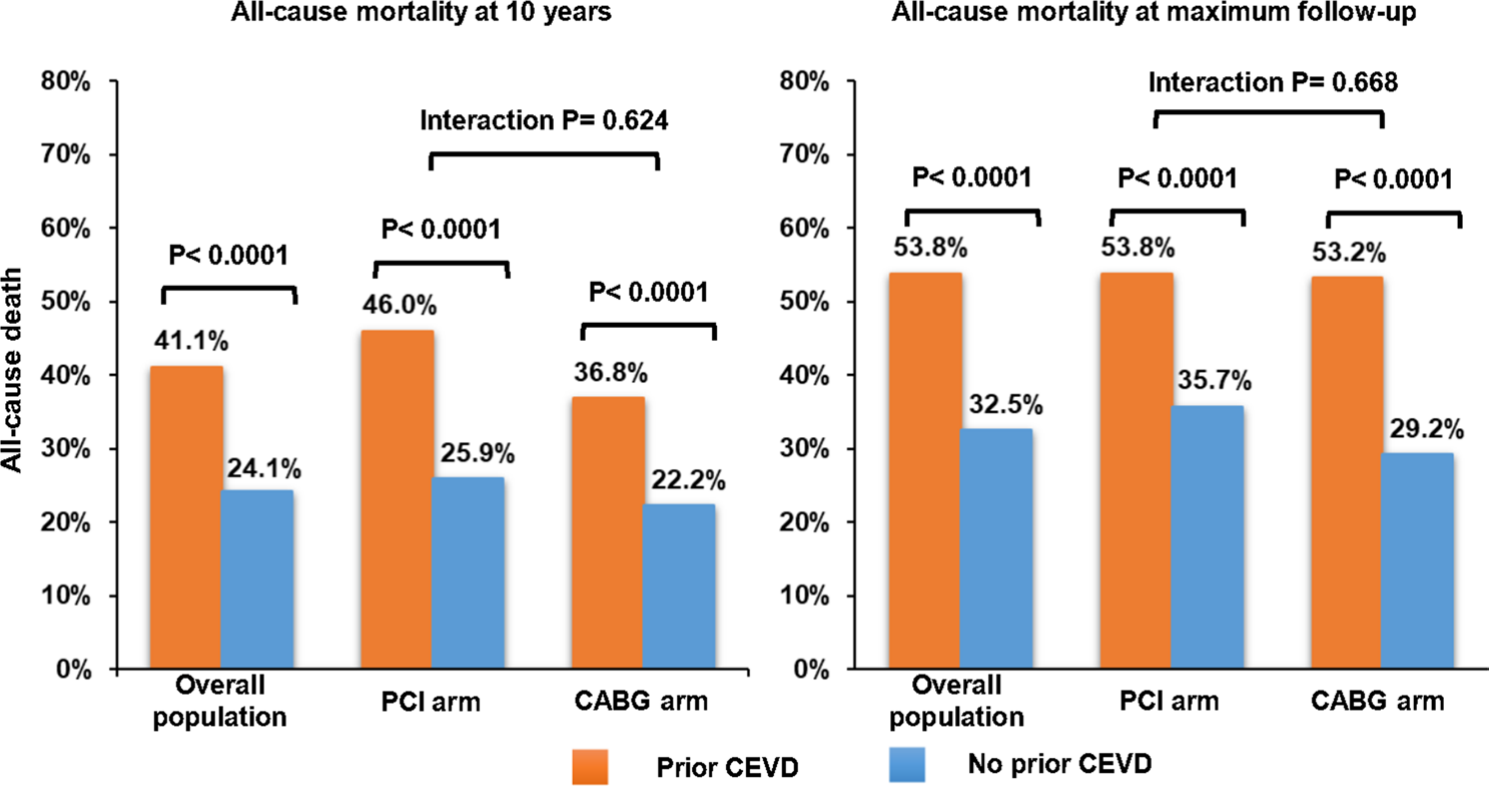

**Supplementary Information:**

The online version contains supplementary material available at 10.1007/s00392-020-01802-x.

## Introduction

The relationship between cerebrovascular disease (CEVD) and coronary artery disease (CAD) has been extensively investigated with numerous studies confirming that CEVD shares common vascular risk factors with CAD [1, 2]. Moreover, the presence of CEVD is associated with worse clinical outcomes after coronary revascularization, and has been reported to be an independent risk factor of long-term mortality in patients with CAD [3–5].

Randomized controlled trials (RCTs) have consistently shown that coronary revascularization by coronary artery bypass grafting (CABG) as opposed to percutaneous coronary intervention (PCI) is associated with an increased risk of stroke [6–8]. In contemporary clinical practice, patients with prior CEVD are often referred for PCI instead of CABG due to concerns from patients and cardiovascular physicians of a higher rate of perioperative stroke and cognitive decline after CABG. Of note, patients with prior CEVD are more likely to have more extensive CAD than those without CEVD, which can lead to poorer clinical outcomes after PCI [3–5, 9]. Therefore, determining the optimal method of revascularization for patients with prior CEVD remains challenging.

To date, there are no data evaluating the impact of prior CVED on long-term (up to 10 years) mortality after revascularization, especially in patients with de novo three-vessel (3VD) and/or left main coronary artery disease (LMCAD). The SYNTAX Extended Survival (SYNTAXES) study established unique 10 year all-cause death in 94% all-comers patients with de novo 3VD and/or LMCAD who were originally randomized to CABG or PCI in the SYNTAX trial [10]. We therefore aim to evaluate the relative benefit of PCI versus CABG in terms of all-cause death at 10 years according to prior CEVD in the SYNTAXES study.

## Methods

### Study design and population

The SYNTAX study design and the primary and final 5 year results of the trial have been published previously [11–13]. In brief, the trial was a prospective, international, multicenter, RCT conducted at 85 centers in Europe and the United States between March 2005 and April 2007. Based on clinical judgment and the consensus of the Heart Team consisting of a cardiothoracic surgeon and an interventional cardiologist and supported by the study coordinator at each center, all-comers patients with de novo 3VD and/or LMCAD in whom clinical equipoise in terms of revascularization strategy between CABG and PCI was assumed, were enrolled and randomized in a 1:1 fashion to either CABG (*n* = 897) or PCI (*n* = 903) with TAXUS Express paclitaxel-drug eluting stents (PES) (Boston Scientific Corporation, Marlborough, MA, USA). The SYNTAX trial (NCT00114972) completed patient follow-up up to 5 years [13]. The SYNTAXES study (NCT03417050) was an investigator-driven initiative that extended follow-up and aimed to evaluate vital status up to 10 years [10]. The extended follow-up was funded by the German Heart Research Foundation (GHF; Frankfurt am Main, Germany). Follow-up was performed in accordance with local regulations of each participating site and complied with the declaration of Helsinki. Informed consent to assess vital status up to 10 year of follow-up was waived by the medical ethical committee.

### Definition of prior CEVD

Prior CEVD was defined as prior stroke, transient ischemic attack (TIA), or carotid artery disease (carotid stent, endarterectomy, known carotid stenosis or bruit without revascularization, or other), which is consistent with a previous report of the EXCEL trial [14]. The presence of prior CEVD was assessed in every patient before randomization by the investigators and collected on the electronic case report form.

### Study endpoints

The pre-specified primary endpoint of the SYNTAXES study was all-cause death at 10 years. The pre-specified secondary endpoint was all-cause death at maximum follow-up. Vital status was confirmed by electronic healthcare record review and national death registry.

### Statistical analyses

All the analyses were performed according to intention to treat principle. The cumulative incidence of clinical adverse events up to 10 years was assessed using the Kaplan–Meier method and compared using the log-rank test. Hazard ratio (HR) with 95% confidence interval (CI) was assessed by a Cox proportional regression model. Multivariate analysis was performed to evaluate whether prior CEVD was an independent predictor of all-cause death at 10 year or the maximum follow-up. The Cox proportional hazards regression model included the following covariates: age, gender, body mass index, hypertension, dyslipidemia, diabetes mellitus, current smoking, peripheral vascular disease, Chronic Obstructive Pulmonary Disease (COPD), impaired renal function (defined as a calculated creatinine clearance < 60 ml/min using the Cockcroft–Gault equation), prior myocardial infarction, the anatomical SYNTAX score and randomized strategy (CABG or PCI). Unfortunately, the relatively small numbers of the specific components of CEVD precluded analysis of the effect of revascularization by type of CEVD.

Continuous variables are reported as mean ± standard deviations (SD) or median and interquartile range (IQR), and were compared using Student’s *t* tests or Mann–Whitney U test, respectively. Categorical variables are reported as percentages and numbers and were compared using Chi-square or Fisher’s exact test as appropriate. All tests are two-sided and a *p* value of < 0.05 was considered to be statistically significant. All analyses were performed using SPSS Statistics, version 25 (IBM Corp., Armonk, 281NY, USA).

## Results

### Study population

**Fig. 1:**
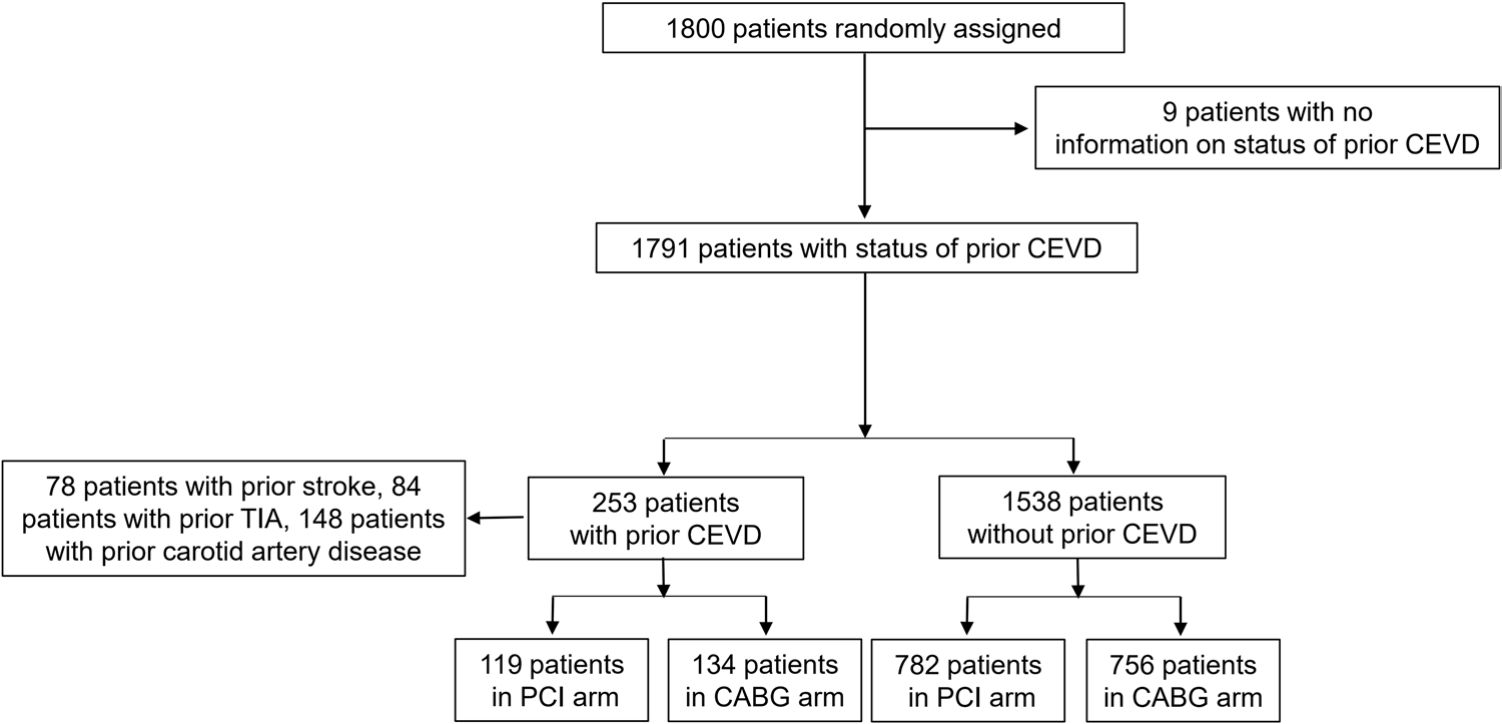
Patient flow diagram of the present study. *CABG* coronary artery bypass grafting, *CEVD* cerebrovascular disease, *PCI* percutaneous coronary intervention, *TIA* transient ischemic attack

In the SYNTAX trial, a total of 1800 patients were randomly assigned to undergo PCI with paclitaxel eluting stents (*n* = 903) or CABG (*n* = 897). The status of prior CEVD was available in 1791 (99.5%) patients who made up the cohort for the present analysis. Among them, 253 (14.1%) patients had prior CEVD (78 patients had prior stroke, 84 patients had prior TIA, and 148 had prior carotid artery disease) (Fig. 1). Vital status at 10 year follow-up was complete in 839 (93%) patients in the PCI group and 841 (94%) patients in the CABG group.

### Outcomes according to prior CEVD

Baseline characteristics according to prior CEVD are shown in Table 1. Patients with prior CEVD were older, had more comorbidities (medically treated diabetes, insulin-dependent diabetes, metabolic syndrome, peripheral vascular disease, chronic obstructive pulmonary disease, impaired renal function, and congestive heart failure), and had a higher EuroSCORE and Parsonnet SCORE, compared with those without prior CEVD. Patients with prior CEVD had more lesions treated compared with those without prior CEVD.

**Table 1:** Baseline characteristics according to prior CEVD

	Prior CEVD (n = 253)	No prior CEVD (n = 1538)	*p* value
Randomization			0.261
PCI	47.0 (119)	50.8 (782)	
CABG	53.0 (134)	49.2 (756)	
Age (year)	68.2 ± 8.7	64.6 ± 9.8	< 0.001
Sex			0.159
Male	74.3 (188)	78.3 (1204)	
Female	25.7 (65)	21.7 (334)	
Body mass index (kg/m^2^)	27.9 ± 4.6	28.0 ± 4.7	0.638
Medically treated diabetes	32.4 (82)	23.9 (367)	0.004
On insulin	15.0 (38)	9.2 (142)	0.005
Metabolic syndrome	43.1 (109)	35.4 (544)	0.041
Hypertension	70.4 (178)	65.7 (1010)	0.144
Dyslipidemia	78.8 (197)	77.8 (1187)	0.720
Current smoker	16.8 (42)	20.8 (319)	0.145
Previous myocardial infarction	35.3 (88)	32.6 (496)	0.392
Previous stroke	31.3 (78)	0 (0)	< 0.001
Previous transient ischemic attack	33.7 (84)	0 (0)	< 0.001
Previous carotid artery disease	58.5 (148)	0 (0)	< 0.001
Peripheral vascular disease	24.5 (62)	7.5 (115)	< 0.001
Chronic obstructive pulmonary disease	14.6 (37)	7.5 (115)	< 0.001
Impaired renal function	32.6 (74)	17.2 (241)	< 0.001
Creatinine clearance (ml/min)	77.0 ± 32.5	87.6 ± 32.6	< 0.001
Left ventricular ejection fraction	57.4 ± 13.1	58.9 ± 13	0.174
Congestive heart failure	7.3 (18)	4.3 (65)	0.036
Clinical presentation			< 0.001
Silent ischemia	22.1 (56)	13.1 (202)	
Stable angina	48.6 (123)	58.5 (899)	
Unstable angina	29.2 (74)	28.4 (437)	
Euro SCORE	5.6 ± 3.0	3.5 ± 2.5	< 0.001
Parsonnet SCORE	11.0 ± 7.6	8.0 ± 6.7	< 0.001
Disease extent			0.489
3VD	58.9 (149)	61.2 (941)	
LMCAD	41.1 (104)	38.8 (597)	
Disease extent			0.509
LMCAD only	3.6 (9)	5.2 (80)	
LMCAD + 1VD	8.3 (21)	7.6 (117)	
LMCAD + 2VD	11.9 (30)	12.2 (187)	
LMCAD + 3VD	17.4 (44)	13.9 (213)	
2VD	1.2 (3)	2.1 (33)	
3VD	57.7 (146)	59 (907)	
Anatomical SYNTAX score	29.9 ± 11.7	28.6 ± 11.3	0.097
Number of lesions	4.6 ± 1.8	4.3 ± 1.8	0.048
Any total occlusion	23.7 (60)	23.1 (353)	0.843
Any bifurcation	73.9 (187)	72.6 (1107)	0.662
Number of stents	4.7 ± 2.2	4.6 ± 2.3	0.639
Total stent length per patient	85.5 ± 45.5	85.7 ± 48.4	0.969
Off pump CABG	16.4 (21)	14.9 (109)	0.668
LIMA use	85.9 (110)	86.0 (629)	0.974
Number of total conduits	2.8 ± 0.7	2.8 ± 0.7	0.495
Number of arterial conduits	1.4 ± 0.7	1.4 ± 0.7	0.497
Number of venous conduits	1.4 ± 0.9	1.4 ± 0.9	0.932
Complete revascularization	58.1 (144)	60.1 (907)	0.543

**Fig. 2:**
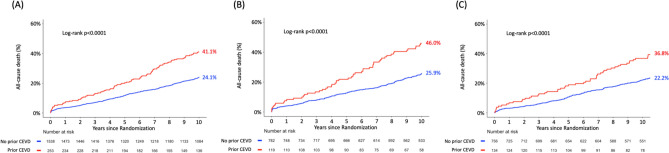
Kaplan–Meier curves for the primary endpoint of all-cause death up to 10 years in patients without (blue) or with prior CEVD (red). **a** Overall population; **b** PCI cohort; **c** CABG cohort. *CABG* coronary artery bypass grafting, *CEVD* cerebrovascular disease, *PCI* percutaneous coronary intervention

The median duration of follow-up was 11.2 years (IQR: 7.7–12.1) overall and 11.9 years (IQR: 11.2–12.4) in survivors. When compared to those without prior CEVD, patients with prior CEVD had a significantly higher risk of all-cause death at 10 years (41.1 vs. 24.1%; HR: 1.92; 95% CI: 1.54–2.40; *p* < 0.001) and at maximum follow-up of 12.6 years (53.8 vs. 32.5%; HR: 1.99; 95% CI: 1.62–2.43; *p* < 0.001) (Fig. 2a, Online Fig. S1A, Table 2).

**Table 2:** Clinical outcomes according to prior CEVD

	Prior CEVD (*n* = 253)	No prior CEVD (*n* = 1538)	HR (95% CI)	*p* value
At 30 days				
MACCE	1.6 (4)	0.6 (9)	1.56 (0.94–2.58)	0.084
Death, stroke, MI	5.1 (13)	4.1 (63)	1.26 (0.69–2.29)	0.448
All-cause death	2.4 (6)	1.2 (19)	1.93 (0.77–4.84)	0.159
Cardiac death	2.4 (6)	1.2 (19)	1.93 (0.77–4.84)	0.159
Any MI	2.0 (5)	3.3 (50)	0.61 (0.24–1.53)	0.293
Any stroke	1.2 (3)	0.6 (9)	2.04 (0.55–7.53)	0.285
Any repeat revascularization	2.8 (7)	2.1 (33)	1.30 (0.58–2.95)	0.522
At 5 years				
MACCE	39.1 (99)	29.9 (460)	1.41 (1.13–1.75)	0.002
Death, stroke, MI	27.3 (69)	16.7 (257)	1.73 (1.33–2.26)	< 0.001
All-cause death	19.4 (49)	11.0 (169)	1.88 (1.37–2.58)	< 0.001
Cardiac death	9.9 (25)	6.3 (97)	1.66 (1.07–2.57)	0.025
Any MI	7.9 (20)	6.2 (96)	1.32 (0.81–2.13)	0.262
Any stroke	4.3 (11)	2.6 (40)	1.74 (0.89–3.39)	0.103
Any repeat revascularization	18.2 (46)	18.6 (286)	1.05 (0.77–1.43)	0.770
At 10 years				
All-cause death	41.1 (100)	24.1 (357)	1.92 (1.54–2.40)	< 0.001
At maximum follow-up				
All-cause death	53.8 (121)	32.5 (442)	1.99 (1.62–2.43)	< 0.001

Data are presented as percentage (number of events). MACCE was defined as a composite of all-cause death, any stroke, any MI, or any revascularization. *MACCE* major adverse cardiac and cerebrovascular events, *MI* myocardial infarction

By multivariate analysis, prior CEVD was an independent predictor of all-cause death at 10 years (adjusted HR: 1.35; 95% CI: 1.04–1.73; *p* = 0.021) and at maximum follow-up of 12.6 years (adjusted HR: 1.45; 95% CI: 1.16–1.82; *p* = 0.001) (Online Tables S1 and S2).

### Clinical outcomes according to revascularization strategy

Among patients with prior CEVD, 119 and 134 patients were randomly assigned to PCI and CABG, respectively. Among 1538 patients without prior CEVD, 782 and 756 patients were randomized to PCI and CABG, respectively (Fig. 1).

Baseline clinical and procedural characteristics according to prior CEVD and revascularization strategies are shown in Table 3. By randomization, baseline clinical and procedural characteristics were largely well balanced between PCI and CABG in patients with and without prior CEVD.

**Table 3:** Baseline characteristics according to prior CEVD and revascularization strategies

	Prior CEVD			No prior CEVD		
	PCI (*n* = 119)	CABG (*n* = 134)	*p* value	PCI (*n* = 782)	CABG (*n* = 756)	*p* value
Age (year)	67.4 ± 8.6	68.8 ± 8.8	0.211	64.9 ± 9.8	64.2 ± 9.8	0.170
Sex			0.202			0.312
Male	70.6 (84)	77.6 (104)		77.2 (604)	79.4 (600)	
Female	29.4 (35)	22.4 (30)		22.8 (178)	20.6 (156)	
Body mass index (kg/m^2^)	28.2 ± 4.8	27.6 ± 4.4	0.343	28.1 ± 4.8	28.0 ± 4.5	0.603
Medically treated diabetes	31.9 (38)	32.8 (44)	0.878	24.6 (192)	23.1 (175)	0.518
Insulin	16.0 (19)	14.2 (19)	0.691	9.0 (70)	9.5 (72)	0.698
Metabolic syndrome	47.9 (57)	38.8 (52)	0.203	35.9 (281)	34.8 (263)	0.304
Hypertension	77.3 (92)	64.2 (86)	0.022	67.5 (528)	63.8 (482)	0.120
Dyslipidemia	78.0 (92)	79.5 (105)	0.760	78.7 (611)	76.8 (576)	0.363
Current smoker	16.0 (19)	17.6 (23)	0.737	18.8 (147)	22.9 (172)	0.049
Previous myocardial infarction	31.6 (37)	38.6 (51)	0.248	32.0 (248)	33.2 (248)	0.643
Previous stroke	29.9 (35)	32.6 (43)	0.651	–	–	
Previous transient ischemic attack	33.1 (39)	34.4 (45)	0.828	–	–	
Previous carotid artery disease	61.3 (73)	56 (75)	0.387	–	–	
Peripheral vascular disease	24.4 (29)	24.6 (33)	0.962	6.8 (53)	8.2 (62)	0.289
Chronic obstructive pulmonary disease	18.5 (22)	11.2 (15)	0.101	6.3 (49)	8.7 (66)	0.066
Impaired renal function	30.4 (34)	34.8 (40)	0.477	18.0 (133)	16.2 (108)	0.377
Creatinine clearance (ml/min)	77.8 ± 32.2	76.2 ± 32.8	0.703	87.9 ± 35.9	87.3 ± 28.5	0.705
Left ventricular ejection fraction (%)	57.5 ± 12.8	57.2 ± 13.4	0.885	59.3 ± 12.9	58.5 ± 13.2	0.357
Congestive heart failure	6.8 (8)	7.8 (10)	0.783	3.6 (28)	5.0 (37)	0.183
Clinical presentation			0.153			0.835
Silent ischemia	16.8 (20)	26.9 (36)		13.6 (106)	12.7 (96)	
Stable angina	51.3 (61)	46.3 (62)		57.8 (452)	59.1 (447)	
Unstable angina	31.9 (38)	26.9 (36)		28.6 (224)	28.2 (213)	
Euro SCORE	5.5 ± 3.1	5.6 ± 3.0	0.731	3.5 ± 2.4	3.5 ± 2.5	0.760
Parsonnet SCORE	10.4 ± 7.6	11.6 ± 7.5	0.217	8.2 ± 6.8	7.8 ± 6.5	0.232
Disease extent			0.430			0.962
3VD	56.3 (67)	61.2 (82)		61.1 (478)	61.2 (463)	
LMCAD	43.7 (52)	38.8 (52)		38.9 (304)	38.8 (293)	
Disease extent			0.756			0.841
LMCAD only	5.0 (6)	2.2 (3)		4.6 (36)	5.8 (44)	
LMCAD + 1VD	7.6 (9)	9.0 (12)		7.4 (58)	7.8 (59)	
LMCAD + 2VD	12.6 (15)	11.2 (15)		12.4 (97)	11.9 (90)	
LMCAD + 3VD	18.5 (22)	16.4 (22)		14.5 (113)	13.2 (100)	
2VD	1.7 (2)	0.7 (1)		1.9 (15)	2.4 (18)	
3VD	54.6 (65)	60.4 (81)		59.2 (463)	58.8 (444)	
SYNTAX score	29.7 ± 11.3	30.0 ± 12.0	0.851	28.3 ± 11.4	28.9 ± 11.2	0.255
Number of lesions	4.6 ± 1.9	4.5 ± 1.7	0.878	4.3 ± 1.8	4.4 ± 1.8	0.477
Any total occlusion	26.1 (31)	21.6 (29)	0.411	23.8 (185)	22.4 (168)	0.514
Any bifurcation	75.6 (90)	72.4 (97)	0.558	71.8 (557)	73.4 (550)	0.469
Number of stents	4.7 ± 2.2	–	–	4.6 ± 2.3	–	–
Total stent length per patient	86.3 ± 45.4	–	–	86.3 ± 48.4	–	–
Off pump CABG	–	16.5 (21)	0.656	–	14.9 (107)	0.651
LIMA use	–	85.8 (109)	0.685	–	86.0 (620)	0.716
Number of total conduits	–	2.8 ± 0.7	–	–	2.8 ± 0.7	–
Number of arterial conduits	–	1.4 ± 0.7	–	–	1.4 ± 0.7	–
Number of venous conduits	–	1.4 ± 0.9	–	–	1.4 ± 0.9	–
Complete revascularization	56.3 (67)	59.7 (77)	0.589	56.8 (440)	63.6 (467)	0.007

Data are presented as mean ± standard deviation or percentage (number). *CABG* coronary artery bypass grafting, *CAD* coronary artery disease; *LIMA* left internal mammary artery, *PCI* percutaneous coronary intervention

Compared with those without prior CEVD, the risk of 10-year all-cause death was higher in patients with prior CEVD both in the PCI arm (46.0 vs. 25.9%; HR: 2.06; 95% CI: 1.52–2.79; p < 0.001) and in the CABG arm (36.8 vs. 22.2%; HR: 1.83; 95% CI: 1.32–2.53; *p* < 0.001) (Fig. 2b, c), and these differences remained significant at maximum follow-up of 12.6 years for PCI (53.8 vs. 35.7%; HR: 1.93; 95% CI: 1.45–2.57; *p* < 0.001) and CABG (53.2 vs. 29.2%; HR: 2.09; 95% CI: 1.57–2.77; *p* < 0.001) (Online Fig. S1b, c). However, the risk of all-cause death at 10 years was similar between PCI and CABG irrespective of the presence of prior CEVD (P-_interaction_ = 0.624) (Table 4).

**Table 4:** Clinical outcomes according to revascularization strategies and prior CEVD

	Prior CEVD				No prior CEVD				
	PCI (*n* = 119)	CABG (*n* = 134)	HR (95% CI)	*p* value	PCI (*n* = 782)	CABG (*n* = 756)	HR (95% CI)	*p* value	*p* value for interaction
At 30 days									
MACCE	9.2 (11)	6.0 (8)	1.57 (0.63–3.91)	0.330	5.4 (42)	4.4 (33)	1.23 (0.78–1.94)	0.378	0.631
All-cause death, stroke, MI	5.9 (7)	4.5 (6)	1.31 (0.44–3.91)	0.623	4.5 (35)	3.7 (28)	1.20 (0.73–1.98)	0.464	0.885
All-cause death	3.4 (4)	1.5 (2)	2.26 (0.41–12.32)	0.347	1.9 (15)	0.5 (4)	3.61 (1.20–10.88)	0.022	0.647
Cardiac death	3.4 (4)	1.5 (2)	2.26 (0.41–12.32)	0.347	1.9 (15)	0.5 (4)	3.61 (1.20–10.88)	0.022	0.647
Any MI	2.5 (3)	1.5 (2)	1.69 (0.28–10.12)	0.565	4.0 (31)	2.5 (19)	1.58 (0.89–2.79)	0.118	0.937
Any stroke	0 (0)	2.2 (3)	0.02 (0–188.75)	0.392	0.1 (1)	1.1 (8)	0.12 (0.01–0.96)	0.046	0.985
Any repeat revascularization	3.4 (4)	2.2 (3)	1.52 (0.34–6.80)	0.583	3.1 (24)	1.2 (9)	2.58 (1.20–5.55)	0.015	0.537
At 5 years									
MACCE	48.7 (58)	30.6 (41)	1.77 (1.19–2.64)	0.005	34.9 (273)	24.7 (187)	1.43 (1.18–1.72)	< 0.001	0.326
All-cause death, stroke, MI	31.1 (37)	23.9 (32)	1.33 (0.83–2.13)	0.243	18.8 (147)	14.6 (110)	1.25 (0.97–1.59)	0.082	0.821
All-cause death	21.0 (25)	17.9 (24)	1.17 (0.67–2.05)	0.579	12.4 (97)	9.5 (72)	1.25 (0.92–1.70)	0.146	0.829
Cardiac death	12.6 (15)	7.5 (10)	1.69 (0.76–3.76)	0.200	8.1 (63)	4.5 (34)	1.73 (1.14–2.63)	0.010	0.954
Any MI	13.4 (16)	3.0 (4)	4.60 (1.54–13.77)	0.006	8.6 (67)	3.8 (29)	2.20 (1.42–3.40)	< 0.001	0.219
Any stroke	3.4 (4)	5.2 (7)	0.63 (0.18–2.15)	0.461	2.0 (16)	3.2 (24)	0.62 (0.33–1.16)	0.135	0.984
Any repeat revascularization	27.7 (33)	9.7 (13)	3.13 (1.65–5.95)	< 0.001	24.2 (189)	12.8 (97)	1.93 (1.51–2.47)	< 0.001	0.156
At 10 years									
All-cause death	46.0 (53)	36.8 (47)	1.33 (0.90–1.97)	0.155	25.9 (195)	22.2 (162)	1.19 (0.97–1.46)	0.104	0.624
At maximum follow-up									
All-cause death	53.8 (58)	53.2 (63)	1.13 (0.79–1.62)	0.502	35.7 (244)	29.2 (198)	1.23 (1.02–1.48)	0.030	0.668

Data are presented as percentage (number of events). MACCE was defined as a composite of all-cause death, any stroke, any MI, or any revascularization. *MACCE* major adverse cardiac and cerebrovascular events *MI* myocardial infarction

### Clinical outcomes according to complexity of coronary artery disease (3VD or LMCAD)

The limited number of events precluded a subgroup analysis according to SYTNTAX score; we performed the analysis according to 3VD or LMCAD. Results demonstrated that rates of all-cause death at 10 years and maximum follow-up were numerically higher after PCI than after CABG but not significantly different in both 3VD and LMCAD patients with prior CEVD (Online Fig. S2).

## Discussion

The SYNTAXES study is the first study to investigate 10­year survival after PCI with drug eluting stents versus CABG in patients with *de novo*3VD and/or LMCAD. The present analysis is the first study to evaluate the potential relative benefit of PCI versus CABG in terms of all-cause death at 10 years according to prior CEVD in stable patients with complex CAD. The main findings of the present study can be summarized as follows: (1) prior CEVD (14.1%) was common among patients with de novo 3VD and/or LMCAD and they had more comorbidities and more extensive CAD compared with those without CEVD; (2) prior CEVD was associated with a significantly increased risk of all-cause death at 10 years; (3) the relative effects of PCI versus CABG on 10 year all-cause death were similar, irrespective of whether patients had prior CEVD or not.

Patients with CAD often have prior CEVD, which itself is associated with a higher prevalence of CAD [1, 2, 15]. Numerous studies have demonstrated that CAD patients with prior CEVD are more likely to have a diffuse, complex and higher disease burden and multiple comorbidities [3–5]. Patients with prior CEVD therefore represent a high risk population and are often excluded from coronary revascularization trials. However, with advances in PCI and CABG techniques, more and more patients with prior CEVD are undergoing revascularization in contemporary practice. In our study, 14.1% of patients who underwent coronary revascularization had a prior history of CEVD, which is comparable to the 12.3% observed in the EXCEL (Evaluation of XIENCE Versus Coronary Artery Bypass Surgery for Effectiveness of Left Main Revascularization) trial [14].

Prior CEVD has been shown to be associated with worse clinical outcomes after coronary revascularization [3–5, 9, 16]. Indeed, we found that prior CEVD was associated with a significantly increased risk of all-cause death at 10 years in both the PCI and CABG arms. These poorer outcomes may most likely be due to the advanced age and presence of a greater number of comorbidities (peripheral vascular disease, chronic obstructive pulmonary disease, impaired renal function) and cardiac risk factors (diabetes, metabolic syndrome) in the CEVD patient cohort (Table 1), some of which were also found to be independent predictors of 10 year all-cause mortality. These observations were further validated by the fact that history of prior CEVD remained an independent predictor of all-cause death at 10 years and at maximum follow-up (12.6 years)even after multivariate adjustment for important clinical confounders (Online Tables S1 and S2).

The optimal revascularization strategy for complex CAD patients with prior CEVD remains unclear. Stroke is one of the most devastating complications after coronary revascularization, leading to a higher risk of mortality and permanent disability [17]. Most previous studies demonstrated that CABG carried a higher rate of stroke, especially in the periprocedural period [6–8, 18]. Hence, in clinical practice, patients with prior CEVD are often referred for PCI instead of CABG. However, recent studies have shown that CABG only increased the risk of perioperative stroke, while the rate of long-term stroke was comparable between PCI and CABG [7, 18–21]. Moreover, as aforementioned, patients with prior CEVD, who have complex and diffuse CAD and multiple comorbidities, and who undergo PCI may experience increased rates of recurrent cerebrovascular events, myocardial infarction, and death9, [16, 22]. It is important to balance the risk of stroke, which represents the major adverse event of CABG, against the risk of other adverse events such as repeat revascularization, myocardial infarction and death, when determining the optimal revascularization modality between CABG and PCI in patients with prior CEVD [23, 24]. Hence, whether high-risk patients with prior CEVD would benefit from PCI rather than CABG is debatable, and there are only limited data supporting this. In addition, intense pre-operative evaluation of patient risk factors, careful assessment of supra-aortic vessels and ascending aorta for atherosclerotic disease, use of off-pump “no-touch aorta” surgery, monitoring of cerebral oximetry for early detection and treatment of cerebral hypoxia, and prevention and treatment of post-operative atrial fibrillation may reduce the risk of perioperative stroke in CABG-treated patients [25, 26].

Recently, Jamie et al. investigated whether high-risk patients with LMCAD and prior CEVD preferentially benefit from revascularization by PCI compared with CABG in the EXCEL trial. They demonstrated that patients with LMCAD and prior CEVD, when compared with those without CEVD, had higher rates of stroke and reduced event-free survival after revascularization, irrespective of the mode of the revascularization. Overall, patients with prior CEVD had higher rates of stroke at 30 days (2.2 vs. 0.8%; p = 0.05) and 3 years (6.4 vs. 2.2%; *p* = 0.0003) and higher 3 year rates of the primary endpoint of all-cause death, stroke, or myocardial infarction (25.0 vs. 13.6%; *p* < 0.0001) [14]. Notably, no data pertaining to the impact of previous CEVD on very long-term (up to 10 years) mortality after revascularization in patients with 3VD and/or LMCAD are available. Not surprisingly, in our present analyses, we demonstrated that prior CEVD was associated with a significantly increased risk of all-cause death at 10 years, with no significant interaction between prior CEVD and revascularization strategy for the relative risk of all-cause death at 10 years. These findings do not support the strategy that patients with prior CEVD should be preferentially referred for PCI rather than CABG. Instead, the heart team [27] should assess the risk/benefit ratio of CABG versus PCI, by considering the periprocedural surgical risk, anatomical complexity, possibility for complete revascularization, potential procedural complications, benefits of each treatment strategy that emerge over time (beyond the periprocedural period), and patient preferences[28] when selecting the optimal revascularization strategy for 3VD and/or LMCVD patients with prior CEVD.

## Limitations

Our findings should be interpreted in light of the following limitations. First, the present study is a post hoc analysis and should be considered as hypothesis-generating only [29]. In the multivariate analysis, a variety of available confounders have been adjusted for, even though, some may exist that may have not been identified. Second, the prior CEVD was site reported and the screening for CEVD was left to the discretion of each physician, which could lead to an underestimation of the rate of CEVD. Third, the number of patients with prior CEVD was relatively small (*n* = 253) and the present subgroup analysis may, thereby, be underpowered [29]. Therefore, further studies with large sample sizes are warranted to compare the relative treatment benefit of PCI or CABG at extended long-term follow-up. In addition, lacking follow-up stroke data and the functional neurological outcomes was another major limitation of the SYNTAXES study. In our current analysis, some less severe CEVD was not included. We only evaluated the impact of the major CEVD on long-term all-cause death, which was consistent with most previous studies, and the major CEVD may more clinical relevant with the long-term outcomes [14]. Finally, the SYNTAX trial was conducted between 2005 and 2007 with use of the first-generation drug eluting stents that were then available for treatment with PCI, which may limit generalizability of our findings to contemporary clinical practice [30]. Nevertheless, the SYNTAXES study, which achieved a relatively high follow-up rate (94%), is the first one to provide randomized data on the 10 year vital status of patients included in the trial [10].

## Conclusions

Presence of prior CEVD in patients with 3VD and/or LMCVD planned for a revascularization procedure represents a high-risk patient group with complex and diffuse CAD and multiple comorbidities. A history of CEVD was associated with a significantly increased risk of all-cause death at 10 years following PCI or CABG. The risk of all-cause death at 10 years in patients having PCI or CABG was not significantly different according to CEVD status. The current findings from the SYNTAXES study do not support preferential referral for PCI rather than CABG in this population on the basis of a history of prior CEVD. Instead, decision making needs to include assessment of both short- and long-terms risks while discussing strategies amongst care providers and with patients.

## Supplementary Information

Below is the link to the electronic supplementary material.

Supplementary file1 (DOCX 719 KB)

## Abbreviations

CABG: Coronary artery bypass grafting
CAD: Coronary artery disease
CEVD: Cerebrovascular disease
LMCAD: Left main coronary artery disease
PCI: Percutaneous coronary intervention
RCTs: Randomized controlled trials
TIA: Transient ischemic attack
3VD: Three-vessel disease
