# Supplementary material for: Ten-year all-cause death following percutaneous or surgical revascularization in patients with prior cerebrovascular disease: insights from the SYNTAX Extended Survival study
Source: Clin Res Cardiol. 2021 Jan 30;110(10):1543–53. doi: 10.1007/s00392-020-01802-x (PMC8484131; doi:10.1007/s00392-020-01802-x)
Supplement: Supplementary file 1 — Supplementary file1 (DOCX 719 KB) [file 392_2020_1802_MOESM1_ESM.docx]

**Online Table 1. Predictors of all-cause death at 10 years**

|  | **Adjusted HR(95% CI)** | **p value** |
| --- | --- | --- |
| Peripheral vascular disease | 2.15 (1.65-2.80) | <0.001 |
| Current smoker | 2.05 (1.58-2.67) | <0.001 |
| Medically treated diabetes | 1.55 (1.25-1.92) | <0.001 |
| Chronic obstructive pulmonary disease | 1.52 (1.12-2.06) | 0.007 |
| Previous CEVD | 1.35 (1.04-1.73) | 0.021 |
| Age (per year increase) | 1.07 (1.06-1.09) | <0.001 |
| Anatomical SYNTAX score | 1.01 (1.001-1.02) | 0.023 |
| Randomization to CABG (instead of PCI) | 0.80 (0.65-0.97) | 0.027 |
| Impaired renal function | 1.22 (0.95-1.57) | 0.116 |
| Previous myocardial infarction | 1.21 (0.98-1.50) | 0.072 |
| Hypertension | 0.98 (0.78-1.22) | 0.849 |
| Sex (male vs female) | 0.94 (0.75-1.19) | 0.621 |
| Dyslipidemia | 0.86 (0.68-1.09) | 0.211 |

CABG: coronary artery bypass grafting; CEVD: cerebrovascular disease; PCI: percutaneous coronary intervention.

**Online Table 2. Predictors of all-cause death at maximum follow-up**

|  | **Adjusted HR(95% CI)** | **p value** |
| --- | --- | --- |
| Current smoker | 2.10 (1.66-2.65) | <0.001 |
| Peripheral vascular disease | 1.98 (1.55-2.52) | <0.001 |
| Medically treated diabetes | 1.62 (1.33-1.97) | <0.001 |
| Previous CEVD | 1.45 (1.16-1.82) | 0.001 |
| Chronic obstructive pulmonary disease | 1.36 (1.02-1.81) | 0.037 |
| Previous myocardial infarction | 1.22 (1.01-1.48) | 0.039 |
| Age (per year increase) | 1.08 (1.07-1.09) | <0.001 |
| Randomization to CABG (instead of PCI) | 0.79 (0.66-0.95) | 0.010 |
| Impaired renal function | 1.21 (0.96-1.51) | 0.099 |
| Anatomical SYNTAX score | 1.01 (0.998-1.01) | 0.124 |
| Hypertension | 1.00 (0.82-1.22) | 0.980 |
| Sex (male vs female) | 0.93 (0.76-1.15) | 0.508 |
| Dyslipidemia | 0.81 (0.66-1.002) | 0.052 |

CABG: coronary artery bypass grafting; CEVD: cerebrovascular disease; PCI: percutaneous coronary intervention.

**Online Fig. 1 Kaplan-Meier curves for the primary endpoint of all-cause death at maximum follow-up in patients with (red) or without prior CEVD (blue) in the overall population (A), the PCI arm (B), and the CABG arm (C)**

**
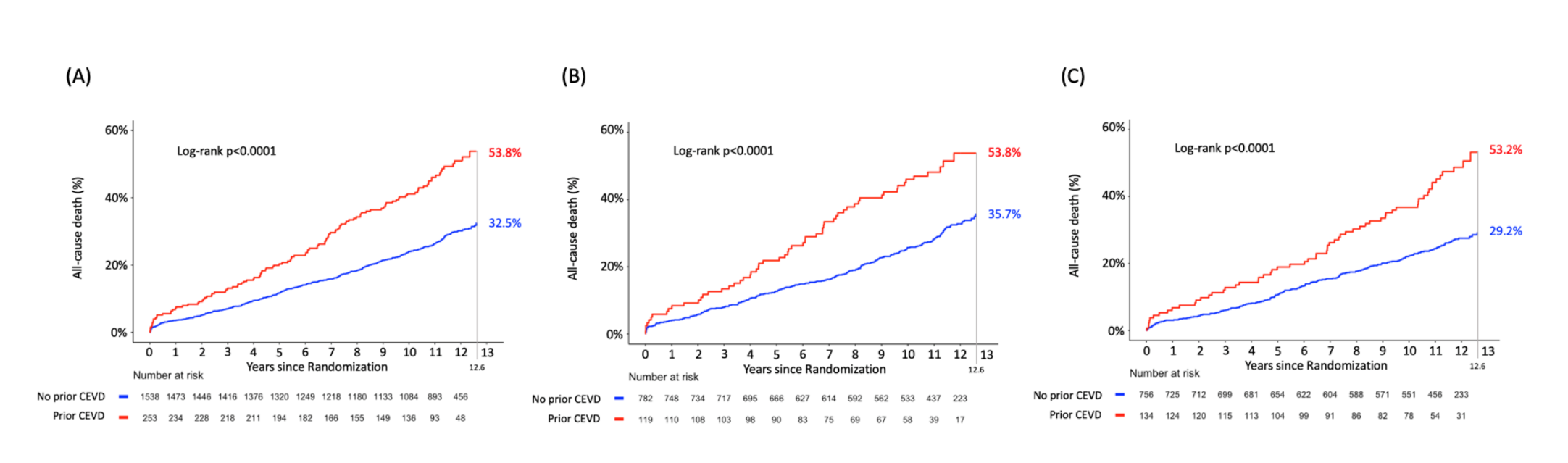
**

CABG: coronary artery bypass grafting; CEVD: cerebrovascular disease; PCI: percutaneous coronary intervention.

**Online Fig. 2 All-cause death at 10-year (A and C) and at maximum follow-up (B and D) for patients with (A and B) and without (C and D) prior CEVD according to 3VD and LMCAD**


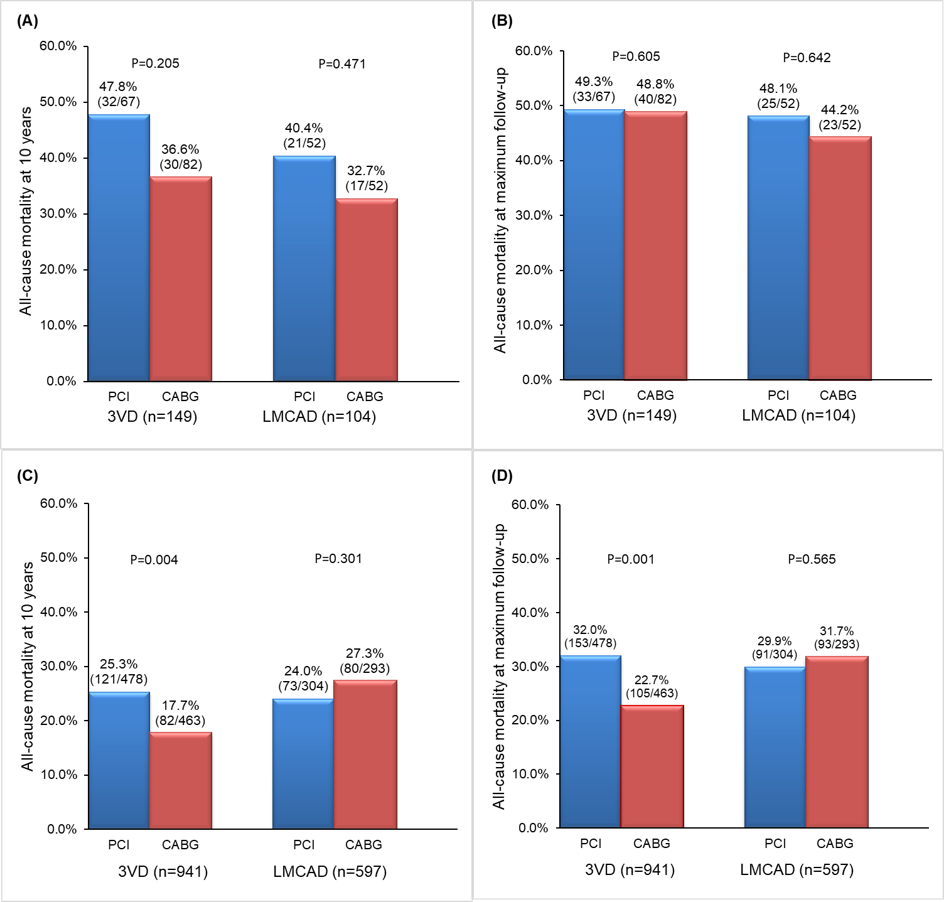


CABG: coronary artery bypass grafting; CEVD: cerebrovascular disease; PCI: percutaneous coronary intervention; 3VD: three-vessel disease; LMCAD**:** left main coronary artery disease.
